# Persistent Hotspots in Schistosomiasis Consortium for Operational Research and Evaluation Studies for Gaining and Sustaining Control of Schistosomiasis after Four Years of Mass Drug Administration of Praziquantel

**DOI:** 10.4269/ajtmh.19-0193

**Published:** 2019-07-08

**Authors:** Nupur Kittur, Charles H. King, Carl H. Campbell, Safari Kinung’hi, Pauline N. M. Mwinzi, Diana M. S. Karanja, Eliezer K. N’Goran, Anna E. Phillips, Pedro H. Gazzinelli-Guimaraes, Annette Olsen, Pascal Magnussen, W. Evan Secor, Susan P. Montgomery, Juerg Utzinger, Joseph W. Walker, Sue Binder, Daniel G. Colley

**Affiliations:** 1 Schistosomiasis Consortium for Operational Research and Evaluation (SCORE), Center for Tropical and Emerging Global Diseases, University of Georgia, Athens, Georgia;; 2 Center for Global Health and Diseases, Case Western Reserve University, Cleveland, Ohio;; 3 National Institute for Medical Research, Mwanza Research Centre, Mwanza, Tanzania;; 4 Center for Global Health Research, Kenya Medical Research Institute, Kisumu, Kenya;; 5 Neglected Tropical Diseases Branch, Centre for Global Health Research, Kenya Medical Research Institute, Kisumu, Kenya;; 6 Unité de Formation et de Recherche Biosciences, Université Félix Houphouët-Boigny, Abidjan, Côte d’Ivoire;; 7 Centre Suisse de Recherches Scientifiques en Côte d’Ivoire, Abidjan, Côte d’Ivoire;; 8 Department of Infectious Disease Epidemiology, London Centre for Neglected Tropical Disease Research, Imperial College London, London, United Kingdom;; 9 Department of Infectious Disease Epidemiology, Schistosomiasis Control Initiative, Imperial College London, London, United Kingdom;; 10 Section for Parasitology and Aquatic Pathobiology, Faculty of Health and Medical Sciences, University of Copenhagen, Copenhagen, Denmark;; 11 Centre for Medical Parasitology, Faculty of Health and Medical Sciences, University of Copenhagen, Copenhagen, Denmark;; 12 Parasitic Diseases Branch, Division of Parasitic Diseases and Malaria, Centers for Disease Control and Prevention, Atlanta, Georgia;; 13 Swiss Tropical and Public Health Institute, Basel, Switzerland;; 14 University of Basel, Basel, Switzerland;; 15 Department of Epidemiology, College of Public Health, University of Georgia, Athens, Georgia;; 16 Odum School of Ecology, University of Georgia, Athens, Georgia;; 17 Department of Microbiology, University of Georgia, Athens, Georgia

## Abstract

Control of schistosomiasis presently relies largely on preventive chemotherapy with praziquantel through mass drug administration (MDA) programs. The Schistosomiasis Consortium for Operational Research and Evaluation has concluded five studies in four countries (Côte d’Ivoire, Kenya, Mozambique, and Tanzania) to evaluate alternative approaches to MDA. Studies involved four intervention years, with final evaluation in the fifth year. Mass drug administration given annually or twice over 4 years reduced average prevalence and intensity of schistosome infections, but not all villages that were treated in the same way responded similarly. There are multiple ways by which responsiveness to MDA, or the lack thereof, could be measured. In the analyses presented here, we defined persistent hotspots (PHS) as villages that achieved less than 35% reduction in prevalence and/or less than 50% reduction in infection intensity after 4 years of either school-based or community-wide MDA, either annually or twice in 4 years. By this definition, at least 30% of villages in each of the five studies were PHSs. We found no consistent relationship between PHSs and the type or frequency of intervention, adequacy of reported MDA coverage, and prevalence or intensity of infection at baseline. New research is warranted to identify PHSs after just one or a few rounds of MDA, and new adaptive strategies need to be advanced and validated for turning PHSs into responder villages.

## INTRODUCTION

The control of morbidity due to schistosomiasis and its eventual elimination has been the focus of multiple World Health Assembly (WHA) resolutions (e.g., WHA 54.19, WHA 65.21, and WHA 66.12)^1–6^ and is being pursued by many neglected tropical disease (NTD) national programs in Africa, Asia, the Middle East, and South America. The standard approach used by most programs is preventive chemotherapy by mass drug administration (MDA)^7^ using the antischistosomal drug praziquantel (PZQ).^8,9^

To provide evidence regarding different ways to control schistosomiasis through MDA, the Schistosomiasis Consortium for Operational Research and Evaluation (SCORE; https://score.uga.edu/) has conducted five studies in four countries (i.e., Côte d’Ivoire, Kenya, Mozambique, and Tanzania) with harmonized protocols to evaluate alternative approaches to MDA. There were originally seven studies in five countries, but two studies in one country had to be redesigned because of protocol deviations in the randomization of study villages. The remaining five studies involved four intervention years, with final evaluation in the fifth year.^10^ In each of the five studies, MDA, either by school-based treatment (SBT) or community-wide treatment (CWT), and whether annual, biennial, or even with two consecutive years without treatment (drug holiday years), reduced prevalence and intensity of infection in the primary outcome group: schoolchildren aged 9 to 12 years. However, as previously reported for some of the studies,^11,12^ the average reduction in prevalence and intensity hide the fact that although many villages demonstrate the expected decreases, others do not, and stubbornly remain what we term persistent hotspots (PHSs). Spatial clusters or hotspots have been recognized in infectious disease epidemiology in various contexts to describe an area of higher burden of disease or higher transmission of disease.^13^ For instance, malaria transmission is known to be higher in hotspots, and it is recognized that elimination of these residual foci will be necessary for malaria elimination.^14–17^ Studies of schistosomiasis programs also show that some communities remain at persistently high prevalence despite MDA.^18–23^ The designation of PHSs intentionally includes the concept of “persistence,” that is, a location that remains at high prevalence and/or infection intensity in the face of consistent preventive chemotherapy with reasonably high coverage.

There is presently no standard definition of PHSs, and, in fact, it is likely that the optimal definition may vary by the data available, the site surveyed, the programmatic goal, or other contextual factors. Previously, we evaluated how four different definitions for PHSs would affect characterization of villages in the SCORE study in Tanzania.^11^ The present study uses one of these definitions—one we believe may be useful in many settings—to determine the proportion of PHSs in all five of the SCORE gaining and sustaining studies, and to evaluate whether the occurrence of PHSs correlates with parameters such as starting prevalence, intensity, and reported coverage. We also examine whether a village’s status as a PHS after 1, 2, or 3 years of MDA predicts its status after 4 years. Given the known focal nature of schistosomiasis transmission and the heterogeneities associated with many vector-transmitted diseases,^24^ the finding of PHSs within these large SCORE studies should, perhaps, not have been unexpected. Nevertheless, the existence and documentation of PHSs indicate the importance of detecting them and adjusting strategies to deal with them to accomplish schistosomiasis control to effectively move to elimination. The objective of this study was to compare and contrast data on PHSs from several different studies to get a clearer and more generalizable understanding of PHSs during MDA treatment for schistosomasis.

## METHODS

### Study data and study arms.

Data from the five large, multicenter SCORE gaining and sustaining studies were used to evaluate PHSs in this article. A description of the overall study, its methods, and baseline data have been published.^10,25–30^ In brief, the sustaining control studies in Côte d’Ivoire and Kenya compared villages that were shown in eligibility testing to have schistosomiasis prevalence of 10–24%. There were three study arms, with 25 villages per arm. Arm 1 received annual MDA by SBT, arm 2 received annual SBT for 2 years followed by two drug holiday years, and arm 3 received SBT every other year. Both of these sustaining control studies focused on the control of *Schistosoma mansoni*.

The gaining control studies were conducted in villages with prevalence ≥ 25% in eligibility testing in Kenya and Tanzania (*S. mansoni*) and Mozambique (*Schistosoma haematobium*). These studies had six study arms, each with 25 villages. Arms 1, 2, and 4 received annual MDA by either CWT or SBT. Arms 3 and 5 received 2 years of MDA followed by 2 years of drug holiday. Arm 6 received MDA every other year. The study diagram is shown in Supplemental Figure 1. All villages had annual measurement of prevalence and intensity, except for years when the village had a drug holiday.

### Study participants and data collection.

The primary study population in these SCORE studies was 9- to 12-year-old children. During the baseline and final study years that are described in this article, 100 children aged 9–12 years were surveyed in each study village. For the annual cross-sectional prevalence and intensity assessment of *S. mansoni* in 9- to 12-year-old children, stool specimens were collected on three consecutive days from each child and eggs were counted on duplicate Kato–Katz thick smears per specimen. For *S. haematobium* prevalence and intensity surveys, two 10-mL aliquots from a single midday urine specimen were filtered, and the filters examined quantitatively under a microscope by two independent experienced laboratory technicians for *S. haematobium* eggs.

### Ethical approval and consent to participate.

Written informed consent was obtained from adults (including parents/legal guardians of children in the study), and assent was obtained from children less than 18 years, except in places where village-level consent is the standard, in which case local requirements were met. Ethical review of research protocol was implemented by human subjects committee in each African country and by the institutional review board (IRB) of their respective northern partners. Studies of gaining and sustaining control of schistosomiasis in Kenya were reviewed and approved by the National Ethics Review Committee of the Kenyan Medical Research Institute (approval numbers SCC 1800 and SCC 1820, respectively) and by the IRB of the CDC (approval no.: 1661). For the study of sustaining control in Côte d’Ivoire, ethical approval was obtained from the ethics committees in Côte d’Ivoire (reference no. 1994MSHP/CNER) and Basel (reference no. EKBB 279/10). In Mozambique, ethical approval was received from the Ministry of Health (reference no. 235/CNBS/10) and the Imperial College Research Ethic Committee (ICREC_10_8_2). In Tanzania, ethical approval was obtained from the National Institute for Medical Research (NIMR; reference no. NIMR/HQ/R.8a/Vol. IX/1022). In addition to these, the University of Georgia IRB implemented an administrative human subject’s review and issued additional approval as per country’s protocol as follows: 10021–0, 10221–0, 10267–0, 10353–0, and 10533–0 for Côte d’Ivoire, Kenya sustaining study, Kenya gaining study, Tanzania, and Mozambique, respectively.

The trials have been registered with the International Standard Randomized Controlled Trial registry under ISRCT numbers 99401114 (Côte d’Ivoire), 14849830 (Kenya Sm1), 16755535 (Kenya Sm2), 95819193 (Tanzania), 32045736 (Niger), and 14117624 (Mozambique).

### Persistent hotspots.

For the analyses herein, we define a PHS as a village that fails to reduce in prevalence by at least 35% and/or fails to reduce in intensity by at least 50% between baseline and year five testing. Although these cutoffs were arbitrarily selected, they were based on the box plots of the percent change in prevalence and intensity in all five studies, shown in Supplemental Figure 2. The reasons for selection of these cutoffs are as follows: it would appear that if an NTD program fails to achieve a one-third decrease in prevalence and/or a one-half decrease in intensity of infection in a given village, then that village deserves additional attention both in regard to the health of its occupants and in terms of achieving program goals, and should be designated a PHS.

### Coverage.

For the present analysis, adequate coverage in both SBT and CWT villages was defined as ≥ 50% reported coverage of school-age children during the 1st year of MDA and ≥ 75% reported coverage in all subsequent years.

### Data handling and analysis.

Data analysis was performed using IBM SPSS Statistics for Windows (Version 25.0; IBM Corp., Armonk, NY). Graphs were generated using GraphPad Prism version 7.0 for Windows (GraphPad Software, La Jolla, CA). Consistent with guidelines put forward by the WHO, *S. mansoni* infection intensity was classified as follows: individuals with < 100 eggs per gram (EPG) were considered to have light infections, those with 100–399 EPG to have moderate intensity infections, and those with ≥ 400 EPG to have heavy infections. For *S. haematobium*, individuals with < 50 eggs/10 mL of urine were considered to have light infection and those with ≥ 50 eggs/10 mL to have heavy infections.^31^ The association between PHSs and two times versus four times MDA, and between PHSs and adequate coverage was assessed using Pearson Χ^2^ tests, whereas the association between PHSs and baseline prevalence of heavy infections was examined using the Mann–Whitney U test. All tests and confidence intervals used the 5% level of significance.

## RESULTS

### Impact of MDA on prevalence and intensity of schistosomiasis.

**Figure 1.**
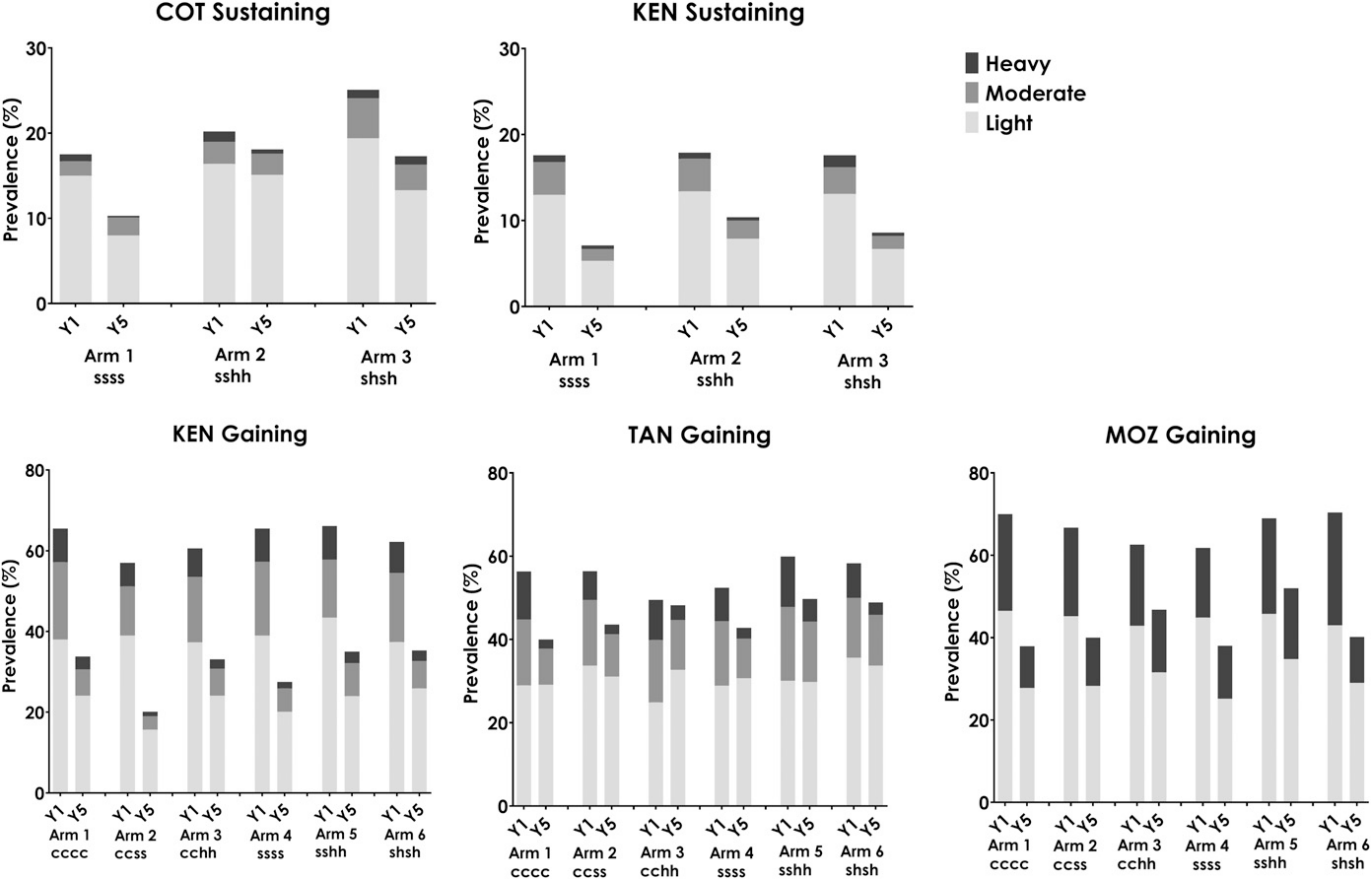
Prevalence by infection intensity category in five Schistosomiasis Consortium for Operational Research and Evaluation studies, by study arm. Data from baseline year (Y1) and final survey (Y5) are shown. In study arm notations, c refers to community-wide treatment, s refers to school-based treatment, and h refers to drug holiday year. χ^2^ *P*-value is < 0.05 for comparisons of Y1 and Y5 in all arms of all studies. COT = Côte d’Ivoire; KEN = Kenya; MOZ = Mozambique; TAN = Tanzania.

In each of the SCORE gaining and sustaining studies, PZQ MDA was administered either as SBT or CWT, and conducted annually or twice during 4 years. After the 4 years, average levels of prevalence and intensity of schistosomiasis among 9–12-year-old children were significantly lower in all study arms (Figure 1).

**Figure 2.**
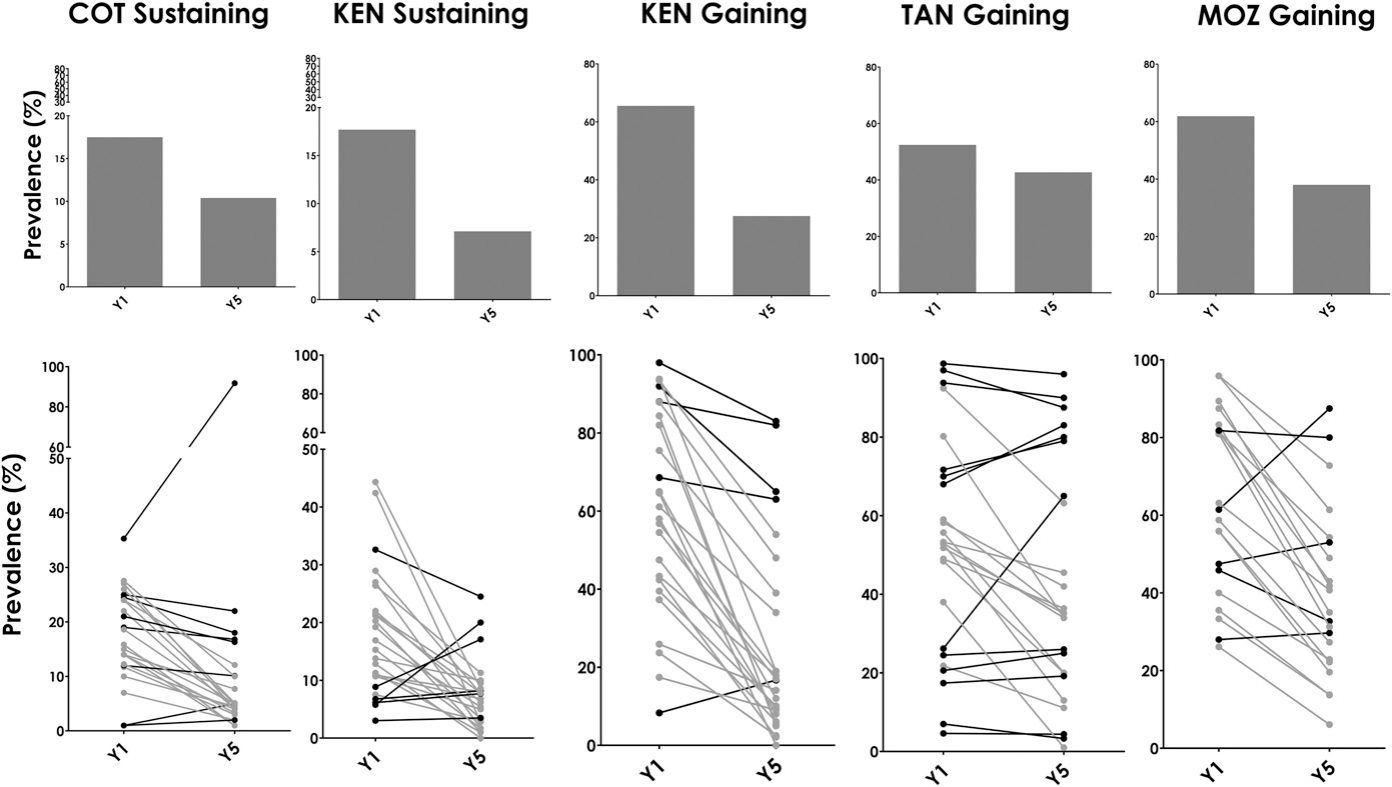
(Top) Mean prevalence at baseline (Y1) and final survey (Y5) in study arms that received 4 years of annual school-based treatment and (bottom) line graphs showing changes in prevalence in individual villages in the same respective study arms. Gray lines depict villages that showed ≥ 35% reduction in prevalence (responders), whereas black lines depict villages with < 35% reduction in prevalence from baseline to year 5 (persistent hotspots based on change in prevalence). COT = Côte d’Ivoire; KEN = Kenya; MOZ = Mozambique; TAN = Tanzania.

However, these averages conceal the fact that there is considerable heterogeneity in the effectiveness of the responses of individual villages among the different MDA regimens. Figure 2 presents an example of this heterogeneity using the study arm that received annual SBT for 4 years, which corresponds to arm 1 of the sustaining studies and arm 4 of the gaining studies. The line graphs (bottom row) in Figure 2 demonstrate the spectrum of change in prevalence among the villages that received 4 years of annual SBT. Eight (32%) of the 25 villages in annual SBT arms of both the Côte d’Ivoire sustaining study and the Kenya sustaining study show only slight decreases or even increases in prevalence over the study period. Increases or slight decreases in prevalences are also seen in the annual SBT arm in five of 25 (20%) villages in the Kenya gaining study, in 16 of 25 (64%) villages in the Tanzania gaining study, and in seven of 21 (33.3%) villages in the Mozambique gaining study. A similar variability in village responses to MDA appeared in every arm in each of all five studies—although most villages demonstrate reduced prevalence by year 5, some decrease only slightly, and some actually increase.

**Figure 3.**
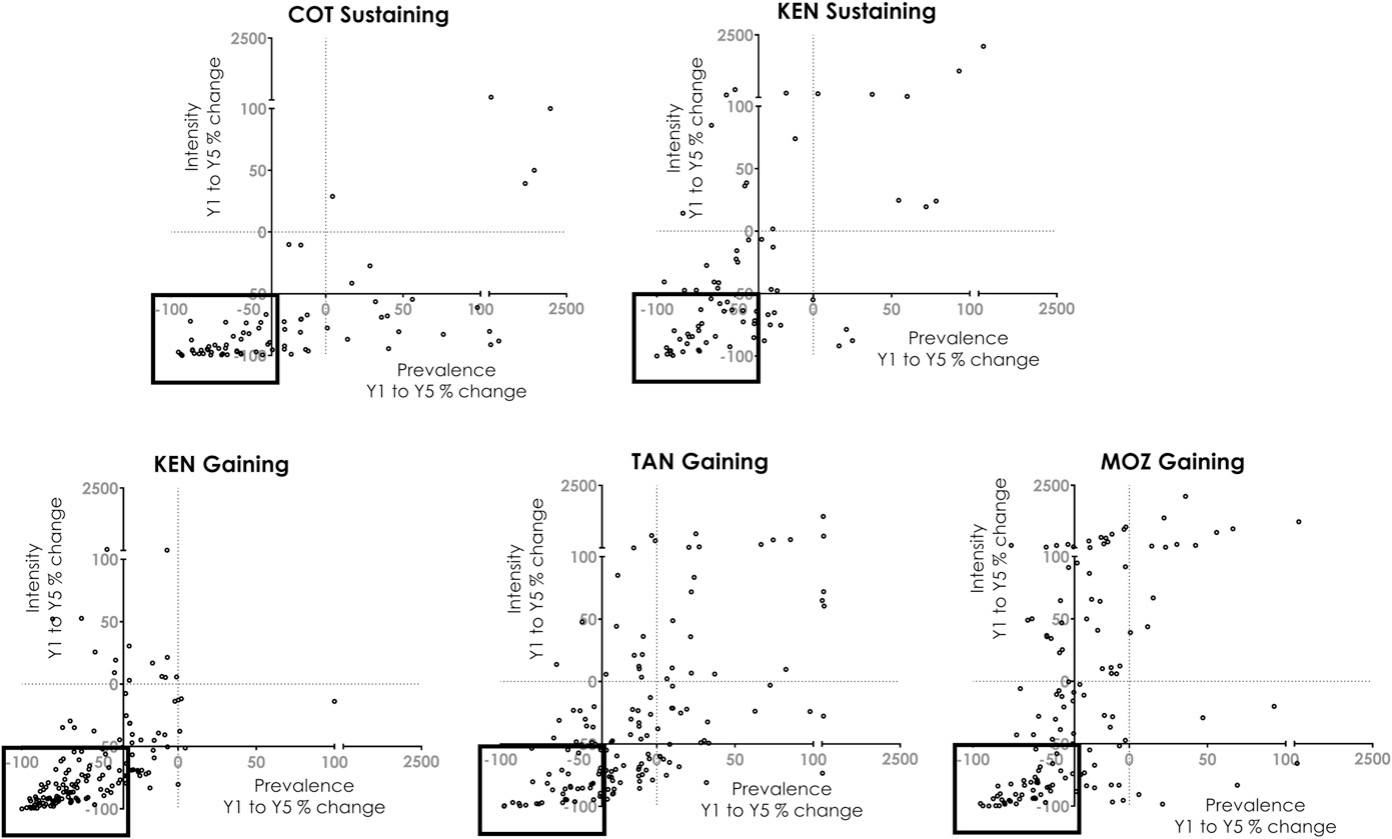
Percent change in prevalence and intensity from baseline (Y1) to final (Y5) survey in individual villages in Schistosomiasis Consortium for Operational Research and Evaluation studies. Each point depicts a study village. The *x* axis shows percent change in prevalence and the *y* axis shows percent change in intensity. Dotted lines are set at *x* = 0 and *y* = 0. The solid lines show the cutoffs for our definition of persistent hotspots (PHSs): Thirty-five percentage reduction in prevalence and 50% reduction in intensity. Dots in the box indicate responding villages, whereas all others are classified as PHSs on the basis of prevalence, intensity, or both. COT = Côte d’Ivoire; KEN = Kenya; MOZ = Mozambique; TAN = Tanzania.

Figure 3 shows scatterplots of the percent change in prevalence (*x* axis) and intensity (*y* axis) from baseline to the end of the study for each individual village in each of the five studies. The dotted lines are set at *x* = 0 and *y* = 0. Villages in the boxes denoted by the solid lines are those considered to be responder villages. Villages in the other three quadrants failed either to demonstrate reductions of 35% in prevalence or 50% in intensity, or both, and are, based on changes in both prevalence and intensity, considered PHS villages. In the Côte d’Ivoire sustaining control study, nearly half (34 of 75, 45.3%) study villages were PHSs and most PHSs (73.5%) met the PHS definition based on prevalence alone, whereas the rest (26.5%) failed to reduce adequately in both prevalence and intensity. In the sustaining control study in Kenya, 41 of 75 study villages (54.7%) were PHSs. Of these, most (41.5%) were classified as PHSs based on their limited reduction in intensity, whereas 36.6% failed to decrease in both prevalence and intensity and 22% failed to decrease in prevalence alone. In the Kenya gaining control study, 35.3% (53 of 150) study villages were PHSs, with 43.4% of PHSs failing in terms of both prevalence and intensity, 35.8% failing in terms of prevalence alone, and 20.8% failing to decrease adequately in intensity. The Tanzania gaining control study had 106 of 148 study villages (71.6%) classified as PHSs, with most PHSs (54.7%) meeting the definition based on limited reduction in both prevalence and intensity, whereas 34% failed to decrease by prevalence alone and 11.3% failed to decrease by intensity alone. The Mozambique gaining study similarly showed a high proportion of PHSs in 87 of 133 study villages (65.4%), with most of these (4.1%) meeting the PHS definition on both intensity and prevalence, whereas 34.5% failed to decrease in intensity alone and 18.4% failed to decrease in prevalence alone.

**Figure 4.**
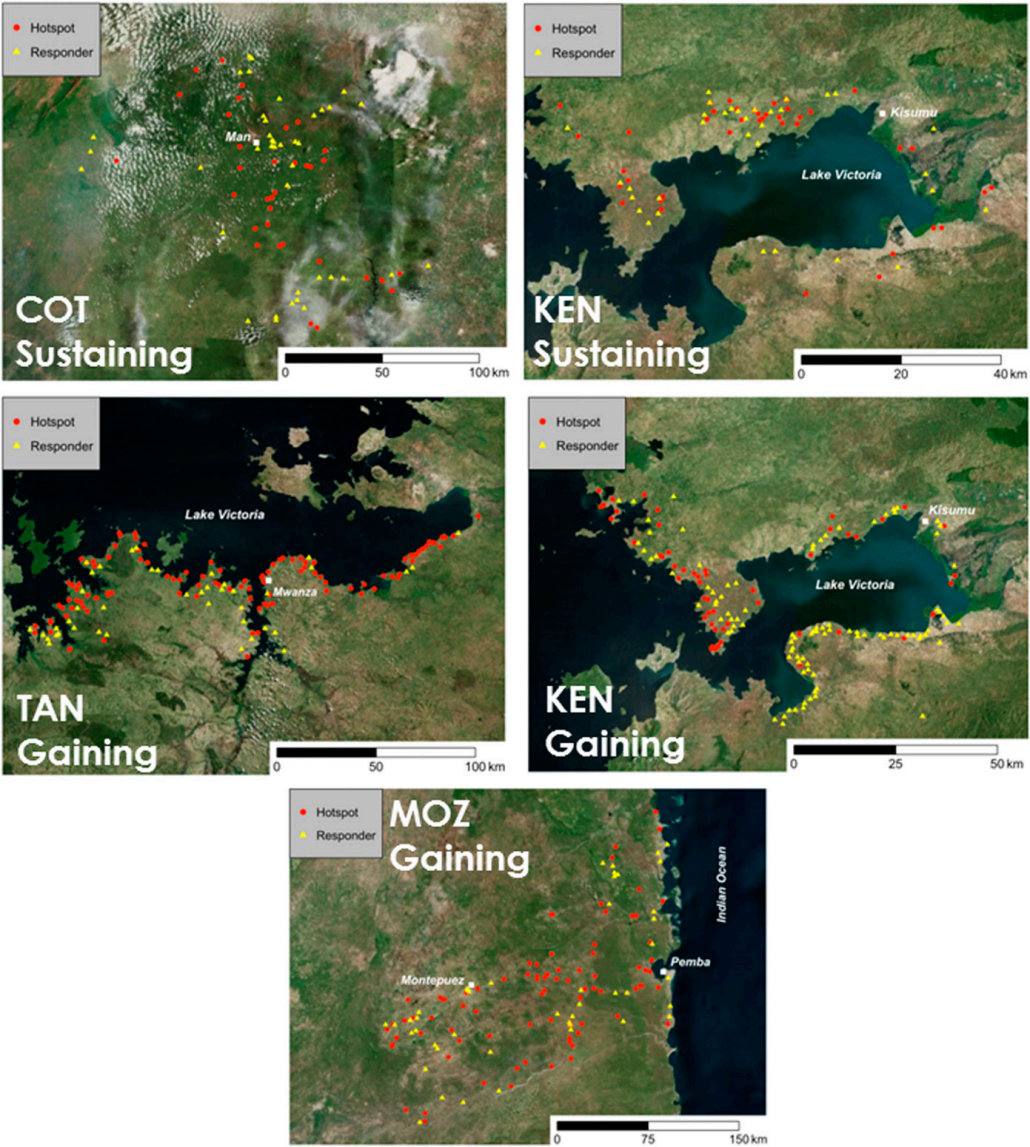
Geographical distribution of persistent hotspots (red circles) and responding villages (yellow triangles) in study areas. Major cities and water bodies are indicated. COT = Côte d’Ivoire; KEN = Kenya; MOZ = Mozambique; TAN = Tanzania. This figure appears in color at www.ajtmh.org.

The geographical distribution of PHSs and responding villages in the five studies is shown in Figure 4. The maps show a spatial clustering of PHSs in the Kenya gaining study, an observation that has been confirmed by geospatial analysis.^12^ By contrast, the Tanzania and Mozambique gaining studies and the Côte d’Ivoire and Kenya sustaining studies show a random distribution of PHSs and responding villages.

**Figure 5.**
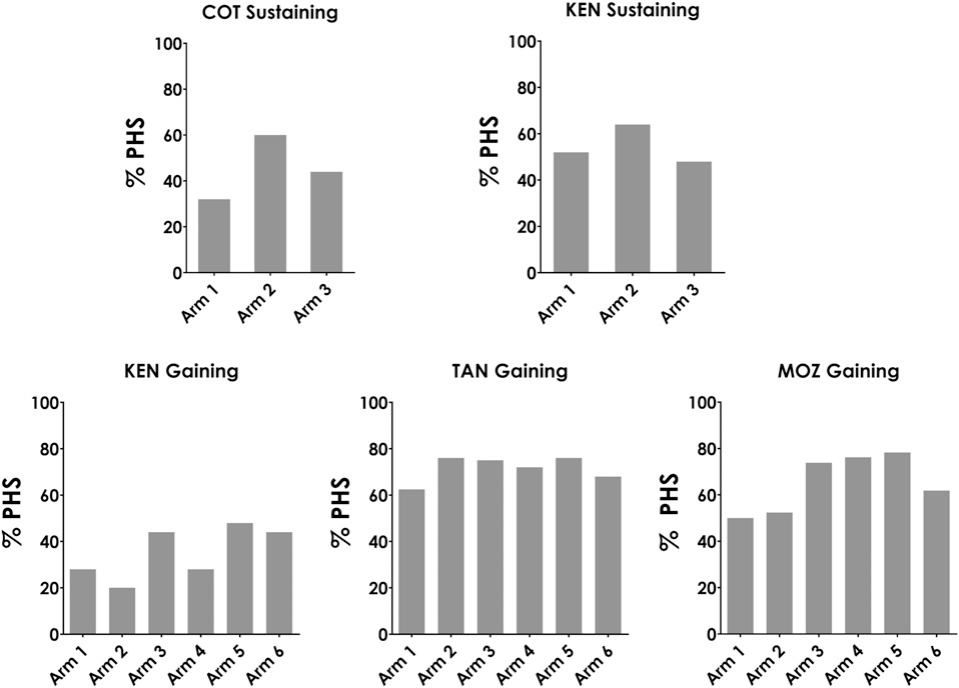
Percent of study villages in each study arm that met the prevalence and intensity definition for persistent hotspots (PHSs) in year 5. COT = Côte d’Ivoire; KEN = Kenya; MOZ = Mozambique; TAN = Tanzania.

Figure 5 presents the proportion of villages, by arm, that meet our definition of PHSs based on both prevalence and intensity of infection at year 5. It can be seen that all arms of the five studies contain PHSs. In the sustaining studies, 30–60% of villages remained PHSs at year 5. There were differences in PHS findings among the gaining studies seen at the end of the study. In Kenya, 20–50% villages were PHSs. In Mozambique, 65–80% of villages were PHSs, but arms with annual CWT or annual CWT/SBT regimens (arms 1 and 2, respectively) had fewer PHSs than the other arms (Figure 4). In Tanzania, in year 5, all arms had more than 60% PHSs, which was higher than expected based on previously reported Tanzania year 4 data.^11^

### Relationship between drug holidays and PHSs.

Although PHSs occurred in all arms in all SCORE sustaining and gaining control studies, study arms having four MDA treatments had fewer PHSs than those that received only two MDAs over the study period. Although the trend is seen in all studies (Table 1), this result was statistically significant only in the Kenya gaining control study (*P* = 0.008) and approaches but does not reach statistical significance in the Côte d’Ivoire sustaining study (*P* = 0.081).

**Table 1:** Comparison of proportion of PHSs in year 5 in the study arms that had mass drug administration (MDA) every year (4×) vs. those that had MDA twice in 4 years (2×)

	PHSs in 2× arms (%)	PHSs in 4× arms (%)	*P*-value
COT sustaining	52.0	32.0	0.081
KEN sustaining	56.0	52.0	0.743
KEN gaining	45.3	25.3	0.008*
TAN gaining	73.0	70.3	0.715
MOZ gaining	71.6	59.1	0.128

COT = Côte d’Ivoire; KEN = Kenya; MOZ = Mozambique, PHSs = persistent hotspots; TAN = Tanzania. χ^2^ *P*-value is indicated.

* Statistical significance (*P* < 0.05).

Further analyses examined the impact of two consecutive MDAs followed by two drug holidays on the number of PHSs. Two MDAs followed by two drug holiday years resulted in more PHSs than MDA without two consecutive drug holidays (Table 2). However, again, the difference was only statistically significant in the Kenya gaining study (*P* = 0.041) (Table 2). This trend was clear but did not achieve statistical significance in the Côte d’Ivoire sustaining study and the Mozambique gaining study (*P* = 0.060).

**Table 2:** Comparison of proportion of PHSs in study arms that had two consecutive drug holidays vs. those that did not

	PHSs in arms with two consecutive drug holiday years (%)	% PHSs in arms that did not have two consecutive drug holiday years (%)	*P*-value
COT sustaining	60.0	38.0	0.060
KEN sustaining	64.0	50.0	0.251
KEN gaining	46.0	30.0	0.041*
TAN gaining	75.5	69.7	0.545
MOZ gaining	76.1	59.8	0.060

COT = Côte d’Ivoire; KEN = Kenya; MOZ = Mozambique, PHSs = persistent hotspots; TAN = Tanzania. χ^2^ *P*-value is indicated.

* Statistical significance (*P* < 0.05).

### Relationship between MDA coverage and PHSs.

**Figure 6.**
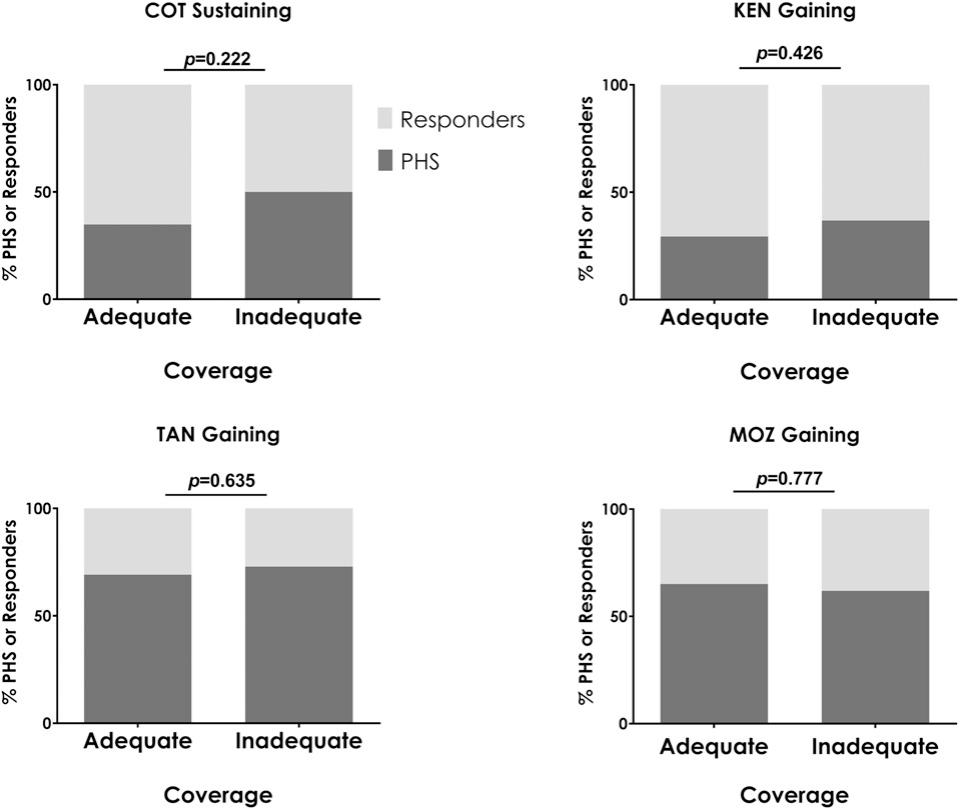
Comparison of proportion of persistent hotspots (PHSs) in villages that had adequate mass drug administration (MDA) coverage throughout the study versus villages that failed to have adequate coverage during one or more MDA. χ^2^ *P*-value is indicated. The Kenya sustaining control study is not included because all villages achieved adequate coverage. COT = Côte d’Ivoire; KEN = Kenya; MOZ = Mozambique; TAN = Tanzania.

One possible explanation for PHSs could be low levels of reported MDA coverage. For these analyses, adequate coverage was defined as ≥ 50% school-age children treated in year 1 and ≥ 75% in subsequent years, for both SBT and CWT villages. All villages in the Kenya sustaining study met the definition of adequate coverage; nevertheless, almost 50% of the villages in each arm were categorized as PHSs (Figure 5). In the other four studies, where this level of coverage was not reportedly uniformly achieved, the proportion of villages that were PHSs was similar, regardless of whether or not a village attained adequate coverage (Figure 6).

### Predicting PHSs based on prevalence and intensity data before year 5.

**Figure 7.**
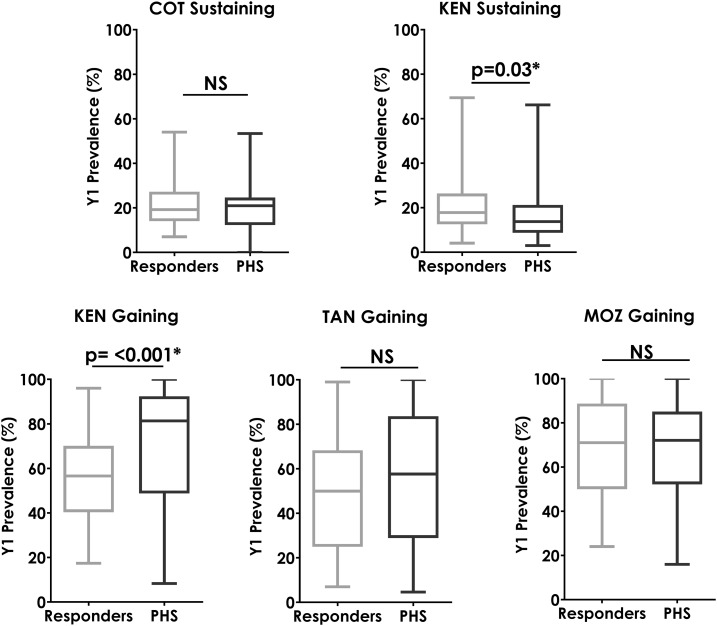
Box plots showing baseline (Y1) prevalence in villages that were responders and those that were persistent hotspots (PHSs) at the end of the study (Y5). Mann–Whitney U-test *P*-values are indicated. COT = Côte d’Ivoire; KEN = Kenya; MOZ = Mozambique; TAN = Tanzania.

Another issue we explored was whether baseline prevalence or intensity was related to the likelihood that a village would respond well to MDA or become a PHS. Figure 7 indicates that the median starting prevalence of PHS villages in the Kenya gaining study was significantly higher than those that became responder villages. Conversely, in the Kenya sustaining study, although the median starting prevalence differed between PHSs and responder villages, the responder villages had the higher starting prevalence. There were no statistically significant differences related to starting prevalence in the studies in Côte d’Ivoire, Tanzania or Mozambique.

**Figure 8.**
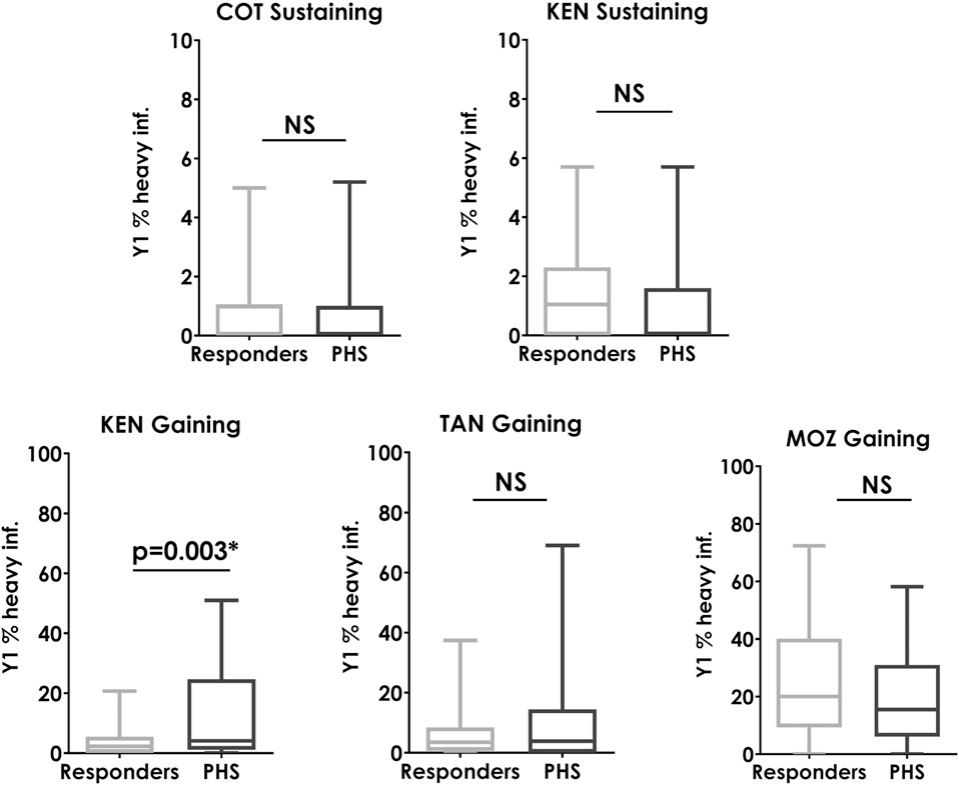
Box plots showing baseline (Y1) prevalence of heavy infections in villages that were responders and those that were persistent hotspots (PHSs) at Y5. Mann–Whitney U-test *P*-values are indicated. COT = Côte d’Ivoire; KEN = Kenya; MOZ = Mozambique; NS = not significant; TAN = Tanzania (*P* > 0.05).

Figure 8 demonstrates that the starting prevalence of heavy infections was a statistically significant predictor that a village would be a PHS only in the gaining control study in Kenya. This relationship was not statistically significant in the other four studies.

**Figure 9.**
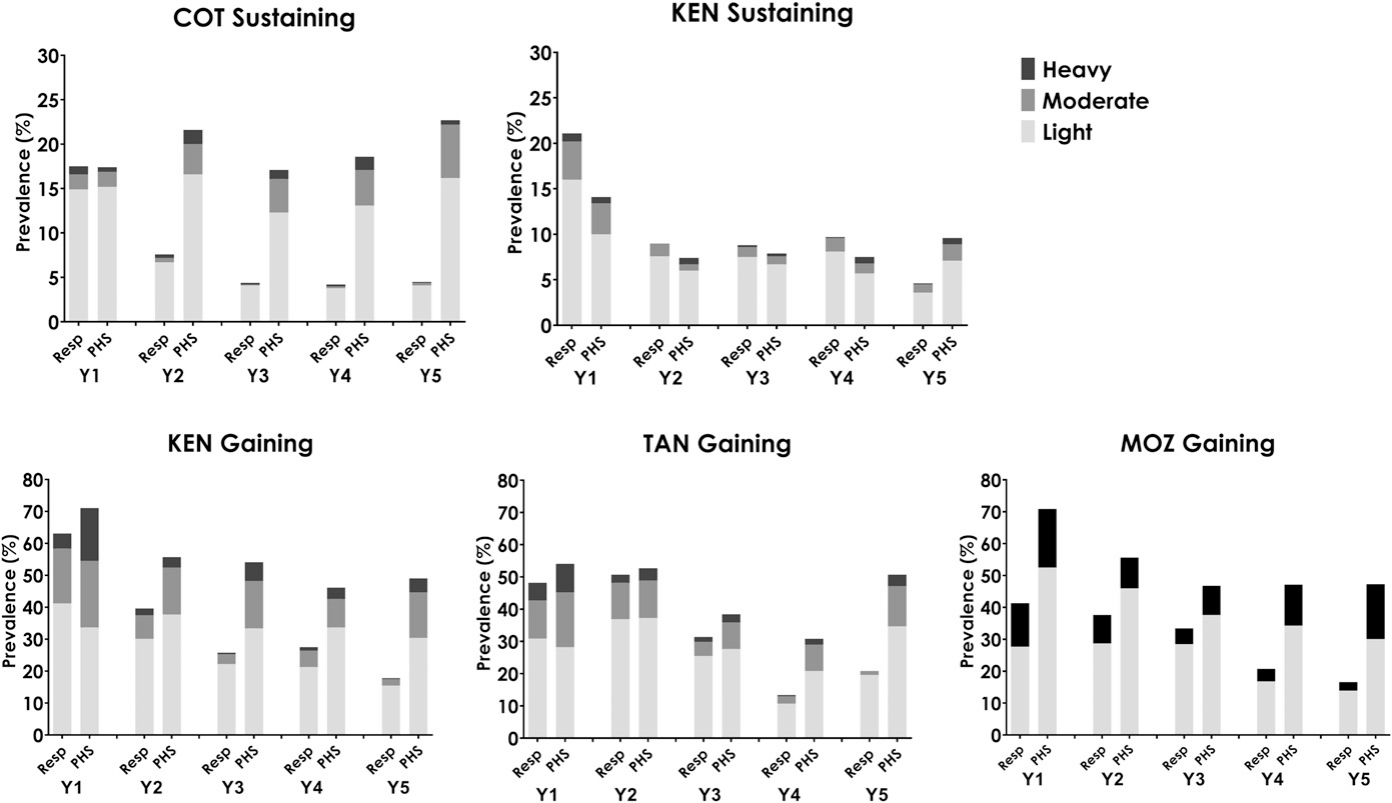
Prevalence and intensity categories for every study year among villages in study arms that received four annual school-based treatments across the 5 years for Schistosomiasis Consortium for Operational Research and Evaluation studies of sustaining and gaining control, stratified into responding villages (Resp) and persistent hotspots (PHSs). COT = Côte d’Ivoire; KEN = Kenya; MOZ = Mozambique; TAN = Tanzania.

We explored whether data from years other than year 1 predicted PHSs by examining villages that received annual SBT—corresponding to arm 1 in the sustaining and arm 4 in the gaining control studies. These study arms were chosen because annual SBT is the only annual treatment regimen implemented in both the gaining and sustaining studies. Prevalence and intensity categories for responding villages and PHSs in annual SBT arms are shown in Figure 9 for all study years and for all studies. For the studies of sustaining control (starting prevalence 10–24%), findings differed by country. In the Côte d’Ivoire sustaining control study, future status could be determined by monitoring after 1 year of MDA, before the year 2 MDA. However, in the Kenya sustaining study, village response before year 5 was not predictive of final status as a PHS, as prevalence was low in both responder and PHS villages in the Kenya sustaining control study in year 2. In the studies of gaining control where starting prevalence levels were ≥ 25%, assessment before the MDA in year 2 or year 3 would likely have predicted which villages would be categorized as responders or PHSs when determined at year 5. Indeed, in Mozambique and Kenya, it seems that a prediction of which villages were to be become PHSs was possible after just 1 year of MDA.

## DISCUSSION

The SCORE studies of sustaining and gaining control of schistosomiasis explored the question of whether different regimens of MDA would lead to different amounts of reduction in prevalence and intensity of schistosomiasis.^10^ The main study finding was that although all regimens decreased overall prevalence and intensity, only a few statistically significant differences were observed between impacts of different regimens.^26,29,32^ However, the observed arm-to-arm decreases represent an average of widely heterogeneous responses to MDA at the individual village level. Thus, although the overall impact of an MDA program may appear satisfactory, many villages (at least 30% in each of these five large studies) may not have appreciable reduction in the prevalence and/or intensity of schistosome infections, and may, therefore, need additional efforts to reduce infection proportions and/or levels. Meanwhile, other villages that make up the average response to any given MDA may have marked reduction in prevalence and intensity after just a year or two of intervention that could perhaps allow adjustments to less intensive MDA in subsequent years.

Our results indicate that PHSs occur both in areas that start out with relatively low prevalence among schoolchildren (10–24%) and those that start out higher (≥ 25%). Whether a village will become a PHS cannot consistently be predicted based on the baseline prevalence or the starting prevalence of heavy infections.

Persistent hotspots occurred in all study arms, even in those with annual CWT. In some cases, there was an indication that four annual MDAs resulted in a lower proportion of PHSs than did two MDAs over 4 years; similarly, in some cases, having MDA at least every other year resulted in fewer PHSs than having two consecutive drug holidays.

Obviously, low MDA coverage can be expected to yield poor outcomes. However, in these studies, the coverage goals were high, nevertheless, achieving these levels of MDA coverage did not seem to be associated with the likelihood of being a PHS. The reliability of some of the coverage data in these studies has been questioned (S. Binder, personal communication); however, reported coverage was generally substantial and coverage data were likely better than would typically be available to most NTD programs. So, the existence of around 30% PHSs after 4 years of MDA with reported high coverage may represent a best-case scenario for ongoing routine MDA programs directed at controlling schistosomiasis.

Based on these analyses, the regimen or frequency of the MDA, the adequacy of MDA coverage, or the overall prevalence or prevalence of heavy infections at baseline does not seem to influence whether villages remain PHSs. It is likely that whether or not a village remains a PHS in the face of MDA-based control rests on the elusive characteristic termed “the force of transmission” at that location. Indeed, modeling studies have suggested that heterogeneous transmission is likely to be a critical determinant of the epidemiology of vector-borne infections.^24^ It is widely acknowledged that schistosomiasis is a focal disease and it is clear that biological amplification through intermediate host snails, open water sources that serve as both people and snail habitat, and socioeconomic realities such as sanitation and human behavior contribute to the force of transmission. It might be expected that village-to-village differences in these parameters could explain differences between PHSs and responding villages in the face of comparable MDA strategies. Presently, several studies are ongoing to determine village characteristics that correlate with being a PHS or a responder village (e.g., R. Musuva, personal communication). In some places, a combination of data types, including infection prevalence and intensity, sanitation, and environmental data, may be predictive of sites that are likely to be PHSs.

Pending the results of ongoing studies of the characteristics that define PHSs versus responder villages, several issues arise that call for efforts by both investigators and NTD control program managers. The first is to determine the earliest time point in an MDA program that will consistently indicate that a given village is not responding as expected and is likely to be a PHS. Current WHO guidelines indicate that programmatic impact assessments of MDAs for control of schistosomiasis be performed after 5 or 6 years.^33^ Although monitoring after 5 or 6 years may be sufficient for diseases such as lymphatic filariasis, our data show that this is not optimal for schistosomiasis: waiting for 5 years would leave many villages with high prevalence and intensity, with consequent effect on the health of residents. It also may result in use of resources in responder villages that do not require the same level of attention, and loss of the opportunity to reallocate resources to areas of greater need. Earlier impact assessments may also require changes in assessment strategies. It is conceivable that sentinel site evaluations or limited sampling strategies would not be sufficient to detect highly focal PHSs and new strategies will need to be developed and rigorously validated in different settings.

The second issue is, once identified, determining what can be done to change a PHS into a responder village. Data such as those reported here suggest that strategies that increase MDA frequency (e.g., from every other year to every year) in PHSs while perhaps decreasing them in responder villages might be possible without worsening overall program outcomes. Comprehensive approaches to PHSs, possibly adding snail control, facilitating sanitation improvements and behavioral change efforts, also need to be tested.

These large, longitudinal SCORE sustaining and gaining studies of MDA implementation in different sub-Saharan African countries have shown, as have prior studies, that MDA does indeed lower the prevalence and intensity of schistosome infections. The size of these SCORE studies has, in addition, made it possible to demonstrate that focusing on the average impact observed obscures many inequities with regard to how individual villages respond to MDA. This finding has implications for what will be needed to achieve even and equitable control and what will be required to achieve the stated ultimate goal of elimination of transmission of schistosomiasis.

## Supplemental materials

Supplemental figures
