# Supplementary material for: Persistent Hotspots in Schistosomiasis Consortium for Operational Research and Evaluation Studies for Gaining and Sustaining Control of Schistosomiasis after Four Years of Mass Drug Administration of Praziquantel
Source: Am J Trop Med Hyg. 2019 Jul 8;101(3):617–27. doi: 10.4269/ajtmh.19-0193 (PMC6726953; doi:10.4269/ajtmh.19-0193)
Supplement: Supplementary file 1 [file tpmd190193.SD1.pdf]

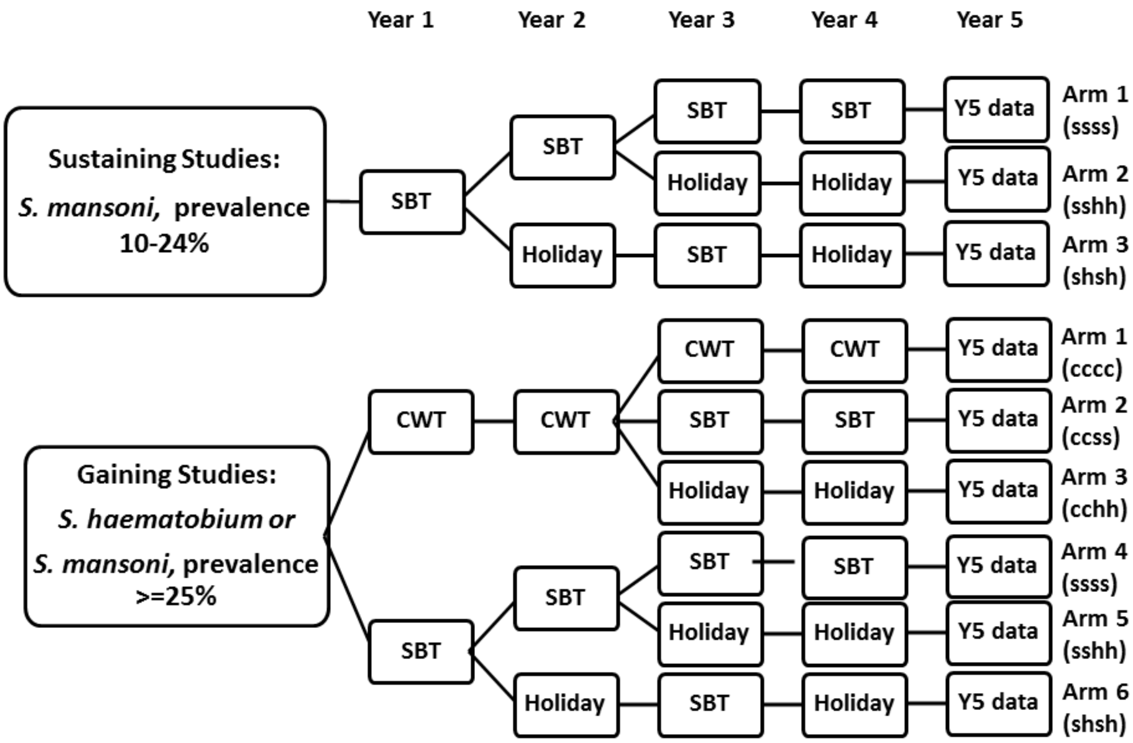

Supplementary Figure 1 Study arms for studies on sustaining control of schistosomiasis in Kenya and Côte ‘Ivoire wherein each arm consisted of 25 villages with prevalence of 10-24% during eligibility testing, and study arms for gaining control studies of schistosomiasis in Kenya, Tanzania and Mozambique wherein each arm consisted of 25 villages with prevalence of ≥25% during eligibility testing. SBT, school-based treatment; CWT, community-wide treatment. Holiday refers to a year in which no treatment was given. Study arms are denoted by codes referring to treatment given over four consecutive years where cccc is annual CWT, ccss is annual CWT for two years followed by annual SBT for two years, cchh is two years of CWT followed by two years of drug holiday, ssss is annual SBT, sshh is annual SBT for two years followed by two drug holiday years and shsh is SBT every other year.

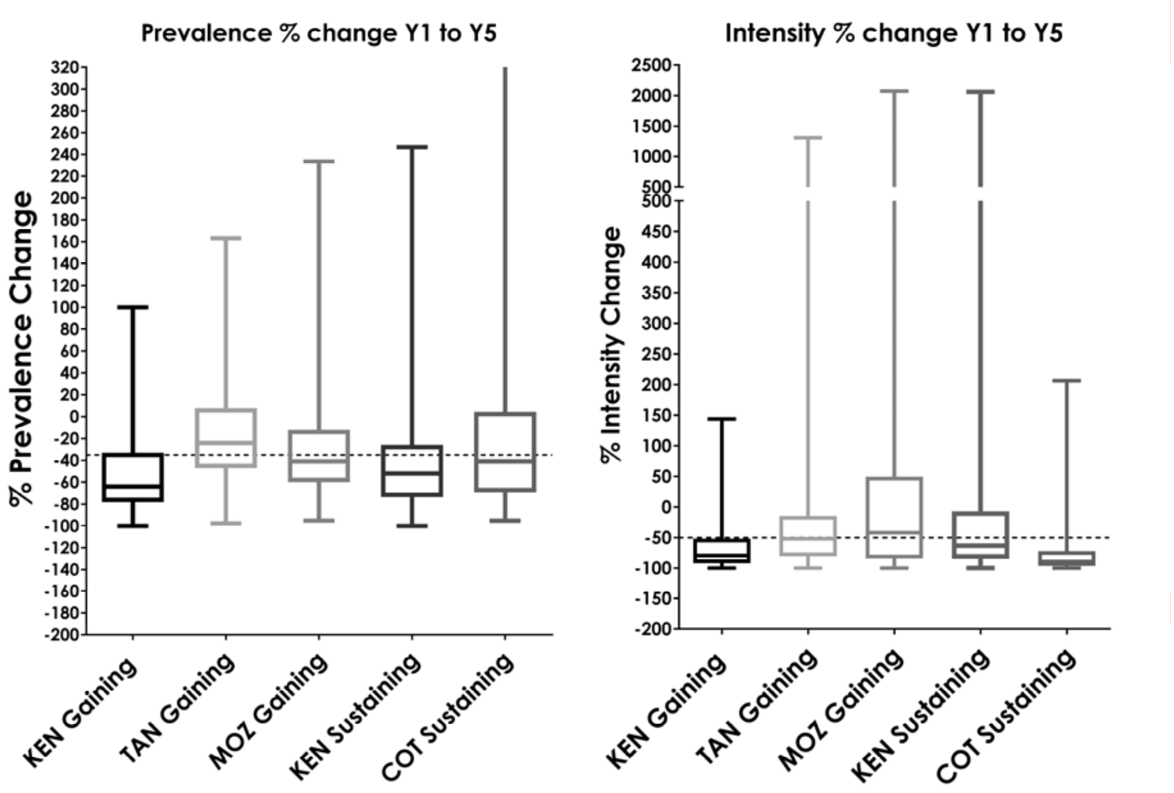

Supplementary Figure 2 Boxplots for each of the SCORE gaining and sustaining studies showing percent prevalence change and percent change in infection intensity from baseline (Year 1) to end of the study (Year 5) in all villages within that study. The box extends from the 25th to the 75th percentile. The horizontal line within the box indicates the median. Whiskers extend from the smallest value up to the largest. Cut offs for prevalence (35% reduction) and intensity (50% reduction) are indicated with dotted lines.
